# A Large Cyst in an Unlikely Location: The Interventricular Septum

**DOI:** 10.1016/j.case.2023.06.001

**Published:** 2023-07-13

**Authors:** George Chalhoub, Andrew Kamel, Jeffrey Levsky, Aldo Schenone, Mario J. Garcia

**Affiliations:** a Department of Internal Medicine, Montefiore Medical Center/Albert Einstein College of Medicine, Bronx, New York; b Division of Cardiology, Montefiore Medical Center/Albert Einstein College of Medicine, Bronx, New York

**Keywords:** Cardiac cyst, Computed tomography, Magnetic resonance imaging, Echocardiography, Interventricular septum

## Abstract

• A large cardiac cyst was discovered in an extremely rare location: the IVS.
• Pre- and postcontrast imaging is essential in the evaluation of a cystic mass.
• When identifying a cardiac cyst, malignancy and infection must be considered.
• The benefit of excising a cardiac cyst is unclear in an asymptomatic older patient.

## Graphical abstract

**Figure:**
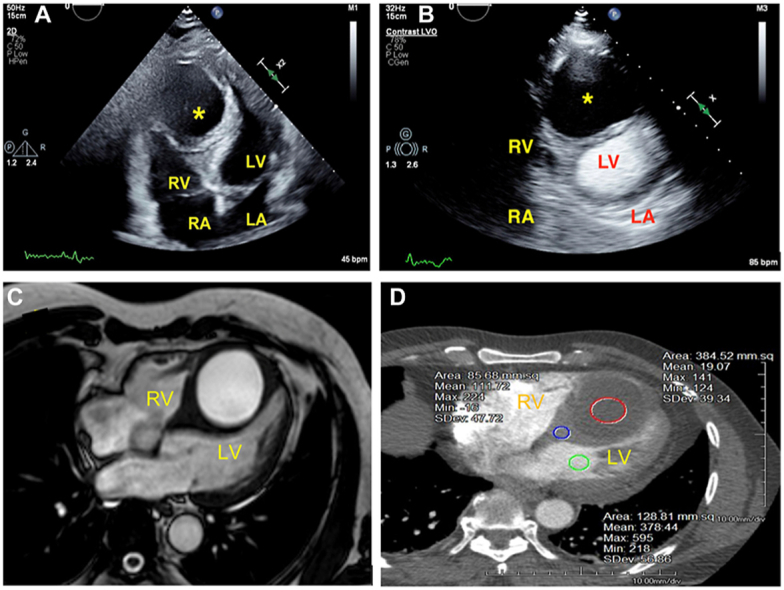

## Introduction

Cardiac cysts are a rare occurrence in adults, with most cysts being congenital, simple in morphology, and benign.^1^ Although complex, septate cysts may indicate echinococcal infections,^2^ simple cardiac cysts are typically pericardial or bronchogenic in origin.^3^ These cysts arise during embryologic development of the pericardial cavity, leading to a preference for the cardiophrenic angles and, less frequently, the cardiac chambers.^4^ The rarest location is the interventricular septum (IVS), with only one documented case in adults after histologic confirmation. We present the case of a 69-year-old man found to have a large, simple cyst in the IVS and the evaluation and management of their care.

## Case Presentation

A 69-year-old man with no known medical history presented to the emergency department with intermittent, pleuritic, substernal chest pain with radiation to the back for 4 days, worse with exertion and resolving spontaneously. They denied any associated fevers, recent illnesses, cough, shortness of breath, weight loss, significant contact with dogs or sheep, cardiac surgical history, and family history of cardiac cysts or cardiac disease. They grew up in Ghana, moved to the United States years ago, and frequently travel back and forth to see relatives. The patient is quite active, walking long distances regularly and climbing upstairs without needing to rest. They take no medications regularly and deny illicit substance use.

**Figure 1:**
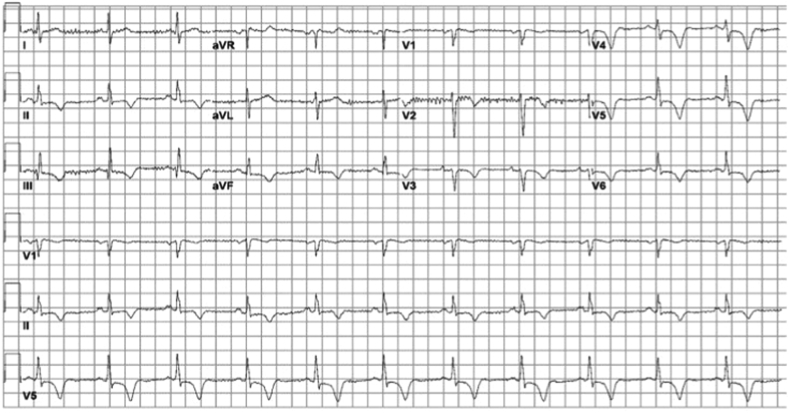
Twelve-lead electrocardiogram demonstrating sinus rhythm at 66 beats/min with T-wave inversions in the inferior and anterolateral leads.

**Figure 2:**
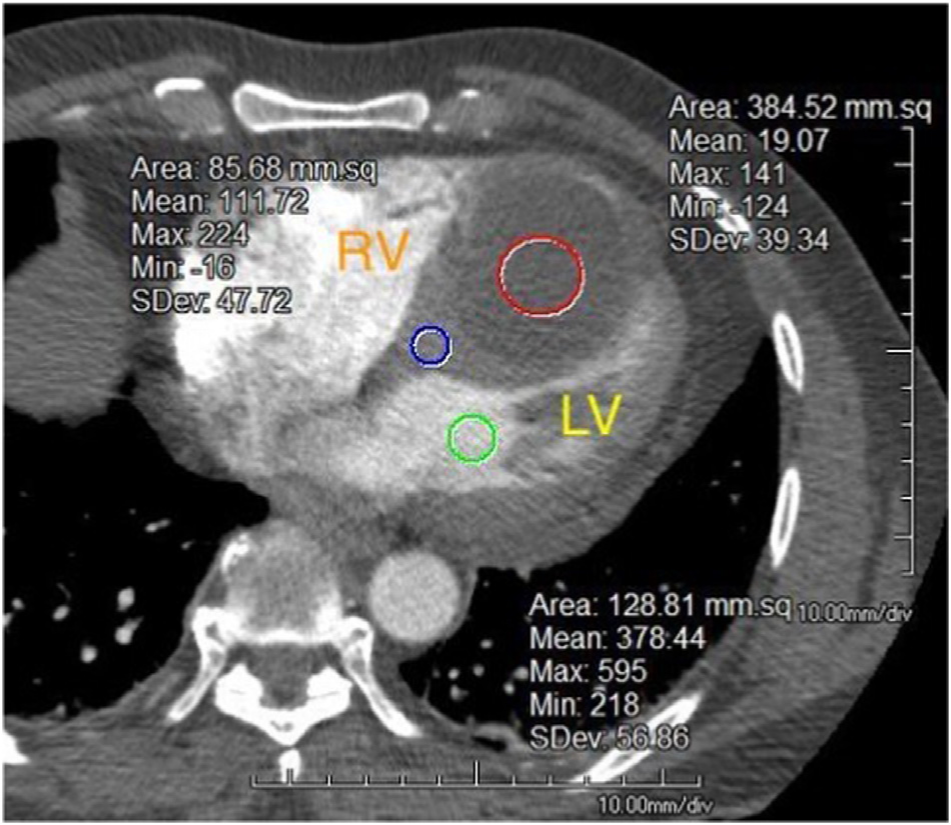
Chest CT, axial view, nongated image obtained after intravenous iodine contrast administration, demonstrating a large interventricular septal mass with low attenuation (*red circle*; mean attenuation 19.07 ± 39.34 Hounsfield units), consistent with avascular fluid content. The mean attenuation values for the IVS (*blue circle*; 111.72 Hounsfield units) and the left ventricle (*green circle*; 378.44 Hounsfield units) are also shown.

**Figure 3:**
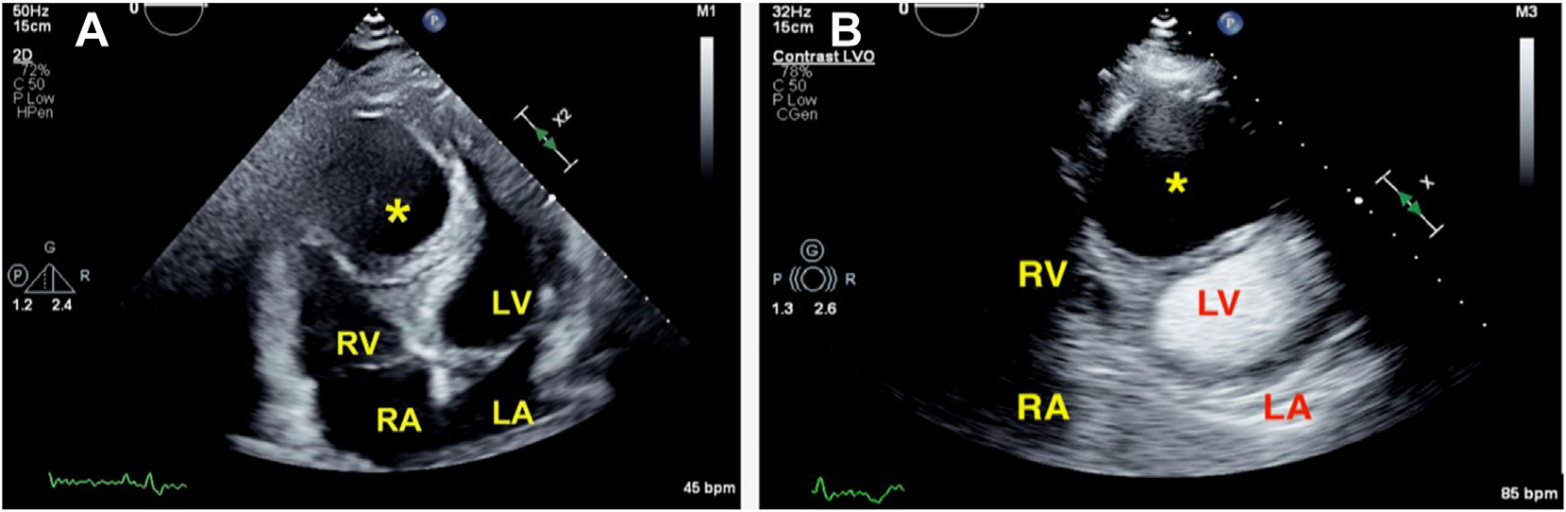
Two-dimensional TTE, apical four-chamber view, systolic phase before **(A)** and after **(B)** the administration of an ultrasound-enhancing agent, demonstrating a large echo-free (cystic) mass (*asterisk*) in the middistal IVS.

In the emergency department, the patient presented with vital signs within normal limits. Electrocardiography showed sinus rhythm at 66 beats/min with T-wave inversions in the inferior and anterolateral leads (Figure 1), though their cardiac enzymes and other basic laboratory values were unremarkable. Chest radiography was performed and showed a small right pleural effusion, but findings were otherwise normal. Computed tomography (CT) with contrast excluded a pulmonary embolism or a pericardial effusion. Instead, it incidentally revealed a 5.0 × 6.0 cm, simple fluid, low-attenuating mass in the IVS without enhancement (Figure 2). Apical four-chamber transthoracic echocardiography (TTE) was then performed and showed a rounded, hypoechoic mass in the IVS, also without enhancement or any continuity between the right and left ventricular cavities following administration of an enhancing agent (Figure 3, Videos 1 and 2). The patient’s chest pain was then treated conservatively with nonsteroidal anti-inflammatory medications given low suspicion for acute coronary syndrome. Given that the patient was from an endemic region, that the cyst’s location was rare, and that early-stage hydatid cysts can appear simple in structure, echinococcus infection was considered and serology was assessed. This was followed by right upper quadrant sonography, which did not reveal any liver abnormalities.

**Figure 4:**
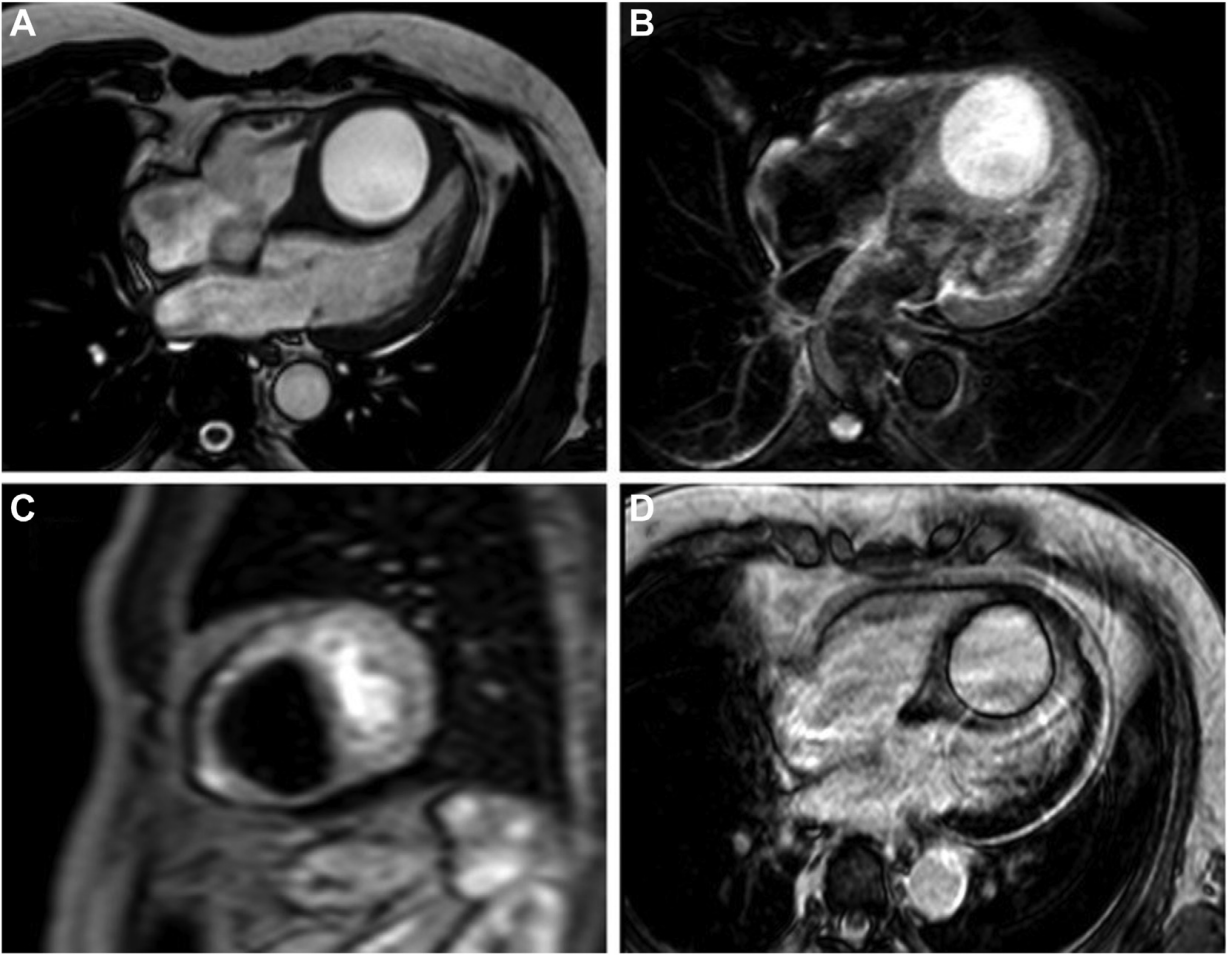
Cardiovascular magnetic resonance images demonstrating a large cyst in the IVS with **(A)** hyperintensity on T1-weighted acquisition, **(B)** hyperintensity on T2-weighted acquisition, **(C)** absent perfusion during intravenous gadolinium administration (short-axis images), and **(D)** no delayed gadolinium enhancement on the thin cystic wall.

To further characterize this mass, cardiovascular magnetic resonance imaging was obtained and showed a 5.5 × 4.8 × 6.0 cm nonenhancing, nonloculated, fluid-filled mass expanding the IVS (Figure 4, Video 3). On the basis of imaging characteristics, history, and laboratory results, the cystic lesion was determined to be benign. The patient was admitted to cardiac telemetry for further monitoring and reported resolution of their chest pain with conservative measures.

After consulting with specialists in cardiothoracic surgery and infectious disease, the primary team concluded that the patient’s cyst was unlikely to be echinococcal but rather benign and possibly congenital in origin. This was based on the significant size of the cyst at the patient’s age, with an absence of any electric conduction abnormalities or hemodynamic instability and the immediate resolution of symptoms without any management of the cyst. The patient was subsequently discharged with a plan for future surveillance imaging. Echinococcal serology were eventually found to be negative.

One month after discharge, the patient was seen in the cardiothoracic surgery clinic and continued to be asymptomatic. They were sent home with a plan for repeat echocardiography in 3 months. At the time of this publication, no follow-up imaging had been performed, although the patient had remained asymptomatic at follow-up appointments.

## Discussion

The differential diagnosis for cardiac cysts is broad, as they can be congenital or acquired from malignancy, trauma, echinococcal or tuberculous infections, fibromatous changes, and ectopic tissue. Among these, pericardial cysts are the most common, accounting for 33% of mediastinal cysts,^3^ with an estimated incidence of 1 in 100,000.^4^ Once discovered on imaging, a cardiac cyst should be evaluated using TTE to evaluate for any interference with chamber inflow and outflow and the cardiac valves. TTE often demonstrates a thin-walled, hypoechoic, circumscribed mass adjacent to the cardiac walls, frequently abutting the right atrium. As such, these cysts can rarely be associated with cardiac tamponade, right main stem bronchus obstruction, or sudden cardiac death of obstructive shock. Any involvement of the IVS raises further concern for impaired cardiac outflow or cardiac conduction abnormalities due to mass effect. The majority have an uneventful natural course.^5^^,^^6^

In the case of our patient, the next major goal was to differentiate whether this mass could be due to malignancy, given that most common cardiac masses are metastatic.^7^ Neoplasms are typically solid, though many can have cystic components or tumor necrosis leading to low attenuation or density on imaging. TTE with an ultrasound-enhancing agent alone is not always adequate to ensure that there is no enhancing soft tissue component. Following up with CT can provide lesion attenuation; this patient’s cyst was shown to have low attenuation. However, it is important to be cognizant that many cysts can have higher than simple fluid attenuation and still not be malignant, making it paramount to include precontrast CT for comparison.

The presentation and management of simple pericardial cysts should be juxtaposed with that of hydatid cysts, which should always be considered in the differential diagnosis of cardiac masses, especially in patients from endemic areas or frequent contact with animals. Hydatid cysts result from infection with the tapeworm *Echinococcus granulosus* during its larval stage. These cysts are described as well-circumscribed and septate, with a predilection for liver and lung involvement. Early-stage hydatid cysts may appear anechoic on sonography and simple on CT or cardiovascular magnetic resonance, similar to this patient’s cyst, but may contain “hydatid sand” that shifts with positioning. This is compared with later stage cysts that contain membranes, daughter cysts, calcifications, or hemorrhage.^8^ They can rarely involve the heart, with an estimated prevalence of 0.5% to 2%.^9^ Distribution of cardiac involvement is dependent on the migratory patterns of the larvae, which travel via blood supply and use coronary blood flow. The left ventricle is most affected (60% of cardiac cases), followed by the right ventricle (10%) and the other chambers at decreasing rates.^10^ The IVS is affected in 4% of hydatid cysts,^11^ as opposed to only one documented case of a simple cyst that could be found in a literature review.^12^ Most cases of IVS hydatid cysts have been in endemic countries, with Turkey accounting for 33%.^10^ Because this patient was from an endemic area (Ghana), echinococcal infection was considered and serology was assessed. The results were ultimately negative, and although this does not rule out echinococcal infection, this patient’s imaging findings were consistent with a benign cyst.

This case also highlights the importance of considering epidemiology, liver imaging, serology, and other clinical data in addition to cyst characteristics on imaging, to arrive at a diagnosis in the absence of confirmatory pathology. This cyst had low attenuation of ∼15 Hounsfield units on CT, making a vascular structure or hydatid cyst less likely, which would tend to exhibit higher densities (25-45 Hounsfield units).^13^ Taken together with the lack of liver involvement, negative echinococcal serologic findings, and the clinical history, the suspicion for hydatid cyst became reasonably low, and the prevailing thought was that this was a benign, likely pericardial cyst.

Given the rarity of cardiac cysts, little has been studied about their management in asymptomatic patients. One retrospective study of 103 patients with pericardial cysts showed that 89% of them were asymptomatic and revealed minor changes in cyst size for a subset of patients who were serially imaged. The investigators concluded that these changes were not clinically relevant and suggested a limited role for serial imaging.^14^ As for the role of aspiration or surgical excision, little evidence exists with respect to the need for, and outcomes of, aspiration and excision in asymptomatic patients.

Considering the resolution of the patient’s presenting chest pain with conservative management, it was considered unlikely that their symptoms were related to this simple cyst. Although diagnostic confirmation as to whether this was a congenital or an acquired cyst was not possible without histopathology, it was deemed harmful and possibly dangerous to obtain tissue confirmation given our patient’s clinical stability and their exceptional functional status at 69 years of age. Our decision to manage this large IVS cyst conservatively has thus far yielded no consequences, as our patient is still asymptomatic at follow-up visits.

## Conclusion

Simple cardiac cysts are rare, benign, mostly incidentally discovered, but occasionally may cause symptoms because of their size and location. We present the case of a large, simple cyst in the IVS of a 69-year-old asymptomatic man, unique for its size and location. Although the cyst’s characteristics increase the risk for cardiac outflow obstruction or conduction abnormality, it is unclear if the benefits of excision outweigh morbidity and mortality in this asymptomatic person. This case illustrates the importance of detailed imaging and management decisions.

## Ethics Statement

The authors declare that the work described has been carried out in accordance with The Code of Ethics of the World Medical Association (Declaration of Helsinki) for experiments involving humans.

## Consent Statement

The authors declare that since this was a non-interventional, retrospective, observational study utilizing de-identified data, informed consent was not required from the patient under IRB exemption status.

## Funding Statement

The authors declare that this report did not receive any specific grant from funding agencies in the public, commercial, or not-for-profit sectors.

## Disclosure Statement

The authors report no conflict of interest.

## Supplementary Data

Video 1 Two-dimensional TTE, apical four-chamber view, demonstrating a large, echo-free (cystic) mass in the distal IVS. Because of the mass effect, both the right ventricle and left ventricle are small.

Video 2 Two-dimensional TTE, apical four-chamber view following administration of an ultrasound-enhancing agent, demonstrating the absence of a connection between the ventricles and cystic mass.

Video 3 Cardiovascular magnetic resonance, sagittal orientation, basal, mid, and distal short-axis (three-slice stack) display during intravenous first-pass gadolinium enhancement, demonstrating absent perfusion (vascularity) of the cystic mass.
